# Optimizing genomic language models for promoter prediction: a comparative study of tokenization and cross-species learning

**DOI:** 10.1093/nargab/lqag025

**Published:** 2026-03-12

**Authors:** Eyal Hadad, Noia Kogman, Lina Golan, Anva Avraham, Reut Ben-Hamo, Zhi Wei, Lior Rokach, Isana Veksler-Lublinsky

**Affiliations:** Faculty of Computer and Information Science, Ben-Gurion University of the Negev, David Ben-Gurion Blvd. 1, Beer-Sheva 8410501, Israel; Faculty of Computer and Information Science, Ben-Gurion University of the Negev, David Ben-Gurion Blvd. 1, Beer-Sheva 8410501, Israel; Faculty of Computer and Information Science, Ben-Gurion University of the Negev, David Ben-Gurion Blvd. 1, Beer-Sheva 8410501, Israel; Faculty of Computer and Information Science, Ben-Gurion University of the Negev, David Ben-Gurion Blvd. 1, Beer-Sheva 8410501, Israel; Faculty of Computer and Information Science, Ben-Gurion University of the Negev, David Ben-Gurion Blvd. 1, Beer-Sheva 8410501, Israel; Department of Computer Science, New Jersey Institute of Technology, Newark, NJ 07102, United States; Faculty of Computer and Information Science, Ben-Gurion University of the Negev, David Ben-Gurion Blvd. 1, Beer-Sheva 8410501, Israel; Faculty of Computer and Information Science, Ben-Gurion University of the Negev, David Ben-Gurion Blvd. 1, Beer-Sheva 8410501, Israel

## Abstract

Large Language Models (LLMs) are increasingly applied to genomic tasks, yet core challenges remain concerning tokenization, evaluation, and data scarcity. This study focuses on promoter classification and systematically evaluates four tokenization methods: non-overlapping 6-mer, overlapping 6-mer, Byte Pair Encoding (BPE), and WordPiece (WPC). We show that the commonly used k-mer approach, specifically the non-overlapping variant, outperforms BPE and WPC across eight organisms, challenging assumptions derived from natural language processing. To ensure robustness, we evaluated performance under two distinct negative data strategies: positive-promoter-shuffled and random-non-promoter-fragments. Using a positional SHAP framework, we demonstrate that the model learns biologically plausible positional patterns rather than exploiting artifacts from these negative data generation processes. Furthermore, evolutionary-informed transfer learning experiments and external validation on an unseen organism reveal that training on phylogenetically related species significantly improves performance, particularly in low-data regimes. These findings underscore the significant impact of tokenization and negative data design, providing practical guidance for refining genomic classifiers.

## Introduction

Large Language Models (LLMs) have become transformative tools across various domains because they generate coherent text and extract meaningful information from extensive datasets. Based on the attention mechanisms [1], LLMs such as Generative Pre-trained Transformers (GPT) [2] and Bidirectional Encoder Representations from Transformers (BERT) [3] have significantly impacted natural language processing (NLP) tasks. Their application, however, is not limited to linguistic domains; LLMs have been utilized in diverse fields like drug discovery [4–7] and genomics [8, 9]. Despite their widespread adoption, unique challenges arise when applying LLMs to genomic data due to the distinct characteristics of biological ‘languages’ compared to human languages. This adaptation of NLP techniques to interpret biological sequences, often referred to as ‘proteome bioinformatics’ when applied to proteins, is a rapidly growing field aiming to decode everything from protein function to disease mechanisms [10, 11].

Genomic sequences differ significantly from human language, primarily in the lack of spaces to outline ‘words’ and the complexity of their multi-dimensional information content [12]. Optimized for human language, traditional tokenization methods may fall short when processing these biological sequences. For instance, subword tokenization algorithms like Byte Pair Encoding (BPE) [13] and WordPiece (WPC) [14] excel in decomposing words into meaningful subunits based on frequency analysis, effectively capturing the morphology of human languages. BPE works by iteratively merging the most frequent pairs of symbols or subwords to create a vocabulary of tokens that effectively represent sequences. The process continues until it reaches a predefined vocabulary size or there are no more frequent pairs to merge, resulting in a vocabulary that includes original characters and newly formed subword tokens. While similar to BPE in starting with individual characters as initial tokens, WPC differs in its approach to merging. Instead of focusing solely on the most frequent pairs, WPC selects token pairs that maximize the likelihood of the entire sequence, given the current vocabulary.

Researchers have adapted tokenization methods tailored explicitly for genomic data to address these differences, such as k-mer tokenization [15]. Widely used in DNA and RNA analysis [16], k-mer tokenization involves breaking down sequences into fixed-length subsequences (k-mers) to create a vocabulary. There are two main variants of this method: overlapping and non-overlapping. In the overlapping approach, each k-mer differs from its neighbor by just one nucleotide, enabling dense representation of local context. In contrast, the non-overlapping approach segments the sequence into adjacent k-mers without redundancy, potentially reducing input length but sacrificing contextual continuity. This tokenization strategy underlies the development of genomic LLMs like DNABERT [17], which leverages k-mer tokens to incorporate nucleotide context and enhance performance on downstream tasks via pretraining on genomic sequences. Models such as miProBert [18] and BERT-Promoter [19] further demonstrate the effectiveness of pretrained models and k-mer tokenization in genomic applications like promoter classification. The successful application of such models is part of a broader trend where NLP-based features are increasingly combined with, or used in place of, traditional biological descriptors to improve predictive power across various biological tasks [20, 21].

The k-mer approach has some limitations in genomic tasks [22], primarily due to its reliance on fixed-length tokens. This drawback can be problematic when dealing with genomic motifs of varying lengths, leading to suboptimal representation. Additionally, k-mers suffer from data sparsity, as the algorithm adds tokens to the vocabulary even if they appear only once, making it challenging for models to learn meaningful patterns, particularly from rare sequences that are not biologically significant. BPE and WPC have been proposed as flexible alternatives to the k-mer approach by allowing for tokens of varying lengths, potentially useful for capturing meaningful biological motifs. Their ability to consider frequency and likelihood during vocabulary building also addresses the data sparsity issue, as they can break down sequences into smaller and more meaningful tokens. These characteristics make BPE and WPC attractive candidates for genomic tokenization. A recent study [23] has begun to explore tokenization strategies for genomic sequences across different model architectures. Existing systematic comparisons have mainly targeted protein sequences [24], emphasizing the broader importance of tokenizer selection in non-human language domains. While many studies focus on k-mer and subword tokenization to capture biological motifs, it is noteworthy that other approaches utilize character-level tokenization. For instance, models such as HyenaDNA [25] and Caduceus [26] have demonstrated strong performance on various genomic tasks using character-level inputs. These models, however, often employ different architectures that are not based on the standard attention mechanism.

Beyond tokenization, evaluating LLMs in the genomic domain has another significant challenge. Unlike human language, where evaluation is relatively straightforward, genomic classification often requires laboratory validation to confirm model predictions. Without laboratory validation, it is challenging to ascertain whether a model has genuinely learned the ‘language’ of genomic sequences or merely exploited artifacts in the negative data. Explainable AI (XAI) tools, such as Shapley Additive Explanations (SHAP) [27], Local Interpretable Model-agnostic Explanations (LIME) [28], integrated gradients (IG) [29], and InterpretML [30], play a vital role in model evaluation by offering insights into model behavior and decision-making processes. XAI is particularly valuable in genomic LLMs, helping to interpret model decisions in genomic classification tasks by highlighting the significance of specific genomic tokens in the classification process [31]. This reliance on computational evaluation is common in promoter-prediction research, as large-scale experimental validation of promoter activity is notoriously challenging [32, 33]. Most studies, therefore, rely on curated promoter annotations from databases such as EPD [34], which aggregates experimentally supported transcription start sites from high-quality sources. These resources serve as the standard reference for benchmarking computational models before more targeted wet-lab experiments can be designed [17, 35].

Another challenge faced by both traditional human language LLMs [36] and genomic LLMs [17] is data limitation. While previous machine-learning (ML) studies have successfully integrated genetic interaction data from closely related organisms based on shared interaction rules [37, 38], leveraging data from other organisms for enhancing genomic LLMs remains unexplored. This idea aligns with the concept of *transfer learning*, a machine learning paradigm in which knowledge gained from one domain or task is transferred to improve performance on a related one [39]. No research has yet utilized genetic sequence data from different organisms to address data limitations in genomic LLMs. While the principle of leveraging evolutionary proximity is established in ML [40], its quantitative impact on data-demanding LLMs in low-data genomic contexts has not been systematically explored. It remains unclear how performance scales with varying amounts of target-species data and at what point the reliance on data from related organisms diminishes.

Promoters are critical elements within the non-coding regions of the genome, playing an essential role in regulating gene expression by controlling the activation or repression of gene transcription. Promoter regions are defined relative to the transcription start site (TSS), the genomic position at which transcription of a gene begins. Many promoters contain specific motifs, such as the TATA box, a well-characterized core promoter element that facilitates accurate recruitment and positioning of RNA polymerase II during transcription initiation. Given the fundamental importance of promoters in gene regulation, researchers have focused on identifying promoter regions, including leveraging LLMs for this task [18, 19].

This study addresses three core challenges in training genomic LLMs for promoter classification: tokenization, evaluation, and data limitations. First, we systematically evaluate common tokenization strategies and reveal that, contrary to expectations from NLP, domain-specific approaches tailored to the continuous nature of genomic sequences, such as non-overlapping k-mer, outperform subword-based methods like BPE and WPC. Second, we introduce an explainability-based evaluation approach to identify the specific sequence regions that drive promoter classification decisions while ensuring that the model has learned meaningful biological features rather than exploiting artifacts in the negative data. Finally, we explore strategies for leveraging cross-species genomic data through distinct pretraining schemes, highlighting the potential of evolutionary-informed training to mitigate data scarcity in underrepresented organisms. This conclusion is further supported by external validation on a species unseen during model training.

## Materials and methods

### Datasets

The datasets used for training and testing the proposed promoter predictor were collected from eight organisms. They contain two distinct classes of promoters: TATA promoters, which include sequences containing the TATA-box, and non-TATA promoters. These datasets were constructed from the Eukaryotic Promoter Database (EPDnew) [41] (downloaded May 2024), a curated, non-redundant collection of eukaryotic POL II promoters where transcription start sites (TSS) have been experimentally verified. For this study, promoter binding sites were defined as 601-nucleotide regions extending from −300 to +300 relative to the TSS. Corresponding negative datasets, consisting of non-promoter sequences of equal length, were generated for each organism as described below. Table 1 shows the number of promoter sequences per organism.

**Table 1.** Summary of the eight datasets used in this study, each corresponding to a different organism. For each dataset, the table includes the organism name, genome version, the number of TATA, non-TATA, and total sequences

Organism	Genome	#TATA	#Non-TATA	#Sequences
*Homo sapiens*	GRCh38/hg38	3065	26533	29598
*(H. sapiens)*				
*Macaca mulatta*	BCM Mmul_8.0.1	631	7701	8332
*(M. mulatta)*	/rheMac8			
*Mus musculus*	GRCm38/mm10	3305	21805	25110
*(M. musculus)*				
*Rattus norvegicus*	Rnor_6.0/rn6	1707	10894	12601
*(R. norvegicus)*				
*Gallus gallus*	Gallus_gallus-5.0	674	5452	6126
*(G. gallus)*	/galGal5			
*Drosophila melanogaster*	BDGP Rel6 +	2598	14372	16970
*(D. melanogaster)*	ISO1 MT/dm6			
*Danio rerio*	Zv9/danRer7	2131	8597	10728
*(D. rerio)*				
*Caenorhabditis elegans*	WS190/ce6	1013	6107	7120
*(C. elegans)*				

#### Negative data

To ensure proper classification between promoter and non-promoter sequences, careful selection of negative datasets is crucial. Two common approaches were frequently used in previous research for constructing negative data in the context of promoters. The first approach, **positive-promoter-shuffled**, is based on random nucleotide substitutions. Inspired by DeePromoter [35] and miProBERT [18], we adopted a uniform perturbation strategy applied consistently across all sequences. In this method, negative sequences are generated by modifying portions of positive sequences. Based on previous research [18] that evaluated various substitution ratios, we divided each positive sequence into 25 subsequences (each was 24 in length). Eight subsequences were randomly selected for substitution and shuffling, while 17 were kept unchanged. Consequently, approximately 68% of each sequence remains unchanged, while the remaining 32% may differ in terms of shared sequence features with the positive dataset, including motifs such as the TATA-box and proximity to the TSS [35]. Given that the overall TATA-box prevalence in the positive data is 12.9% (ranging from 7.6% to 19.9% across organisms), the expected TATA frequency in the negative set is therefore 8.8% (Table 1). It is noteworthy that other studies, such as GROVER [16], have employed a much higher shuffling ratio of 75%. Our choice of 32% perturbation rate was based on the systematic analysis in miProBert [18], which highlighted substitution ratios in the range of 28–36% as optimal for generating biologically plausible negative sequences.

The second, **random-non-promoter-fragments** approach, selects 601 base pair sequences from the same chromosome as the corresponding positive sequence. Following the general concept used in studies like BERT-Promoter [19], we implemented a strict negative generation protocol where new negative sequences are generated by choosing a random TSS from the same chromosome, ensuring a distance of at least 600 nucleotides from any positive TSS or previously generated negative TSS to avoid overlap. This process is repeated for each positive sequence, aiming to maintain a balanced dataset for training and testing.

We acknowledge that generating an ideal negative dataset is a fundamental challenge in promoter prediction, as experimentally verified non-promoter sequences are generally unavailable. Therefore, our choice to employ two distinct methods was a deliberate strategy to ensure the robustness of our findings. These two approaches, random-non-promoter-fragments and positive-promoter-shuffled, represent the primary strategies used in the field [18, 19, 35]. By validating our models across both methods, we aimed to mitigate the inherent limitations of any single approach. We hypothesize that such frameworks, despite relying on inferred negative data, allow models to learn biologically meaningful distinctions, detecting characteristic patterns that differentiate genuine promoters from artificial or background-like sequences, a principle successfully applied in similar regulatory sequence prediction tasks such as microRNA-target interaction [42].

#### Train-validation-test split

To prevent data leakage in our train-validation-test split, we first applied the CD-HIT clustering technique [43] to the positive sequences from all organisms. We set a sequence identity threshold of 80%, a standard practice for effectively removing redundancy while preserving biological diversity. This threshold represents the minimum similarity supported by the CD-HIT software for nucleotide sequences. It has been used in similar large-scale DNA/RNA analyses [44], making it a well-established baseline. All sequences within a given cluster were assigned exclusively to the training, validation, or testing set, ensuring no cluster was split between them. To create the train-validation-test partitions, we employed a size-stratified accumulation strategy. Clusters were sorted by size in ascending order. We first populated the testing set (targeting 20% of the data) with the smallest clusters (primarily singletons), followed by the validation set (targeting 15%). The remaining clusters, including all large families and any residual small clusters, were assigned to the training set (approximately 65%). This ‘bottom-up’ approach ensures that the model is evaluated on the most unique and challenging examples (singletons), while the training set benefits from the diversity of larger sequence families. Consequently, the exact split ratios varied slightly across organisms depending on their specific cluster distributions (see Supplementary Fig. S1).

**Figure 1.**
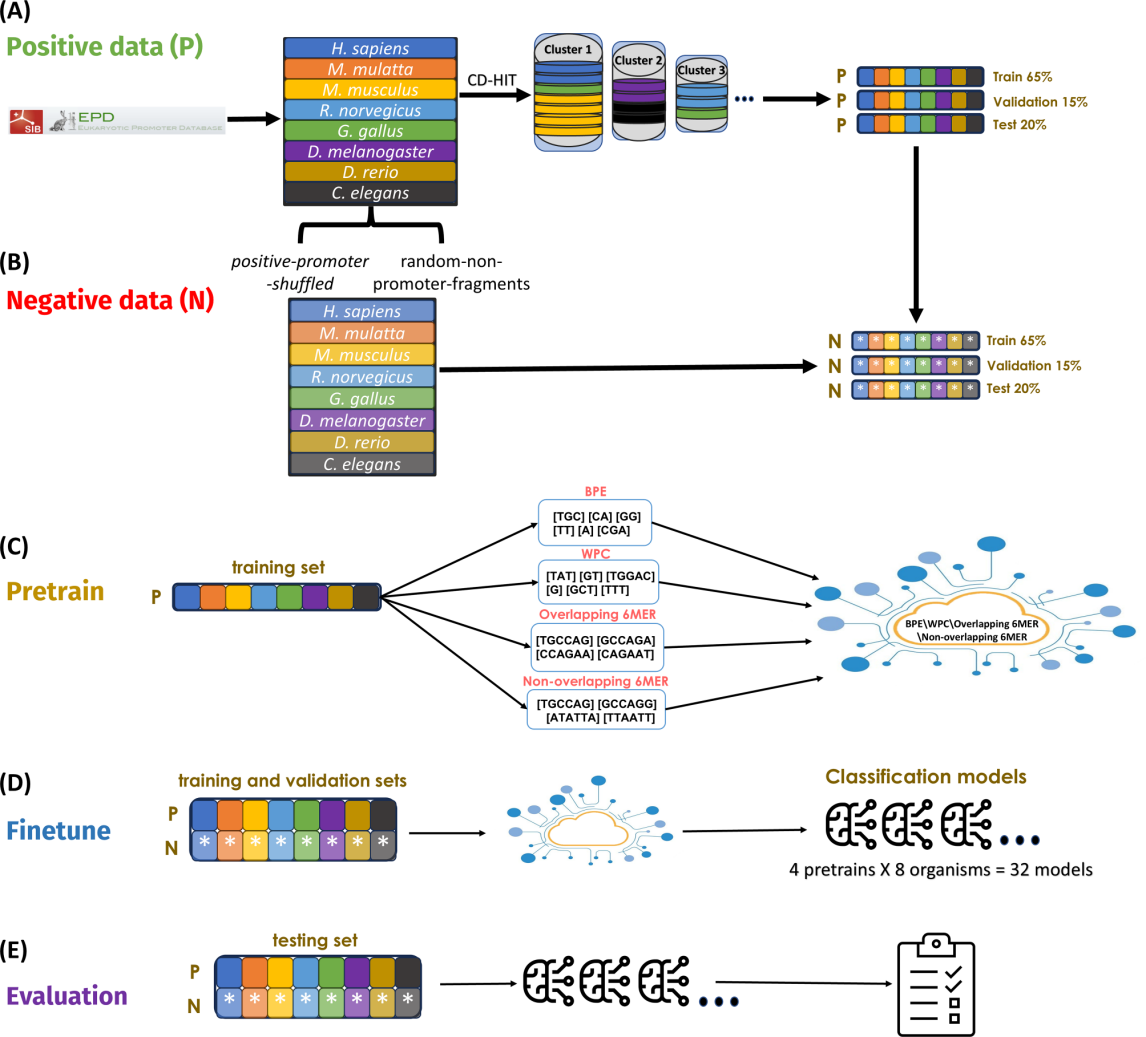
Outline of the complete five-stage experimental workflow used in this study. **(A)** Positive data preparation: Positive promoter sequences from eight organisms (e.g., *H. sapiens, M. musculus*, etc.) were collected from the EPD repository. Clustering with CD-HIT was applied to the positive dataset to group sequences with at least 80% similarity. The dataset was then split into training (65%), validation (15%), and test (20%) sets. **(B)** Negative data preparation: For each positive sequence, corresponding negative sequences were generated using the positive-promoter-shuffled and random-non-promoter-fragments methods, resulting in the same 65%/15%/20% split. **(C)** Pretrain: In the pretraining stage, four tokenization methods: BPE, WPC, overlapping 6-mer, and non-overlapping 6-mer, were applied to the positive training set to create tokenized inputs. Each tokenized dataset is used to train a masked language model, resulting in four pretrained models. **(D)** Finetune: In the finetuning stage, each pretrained model is finetuned on labeled datasets specific to one of the eight organisms, using both the positive and negative training and validation sets. This produces 32 classification models (4 tokenization methods × 8 organisms). **(E)** Evaluation: In the evaluation stage, each of the 32 classification models is evaluated on a held-out test set (containing both positive and negative sequences) to assess performance.

The analysis was performed on a single train-validation-test split, a methodological decision driven by our CD-HIT clustering. Generating alternative splits would have required including sequences from larger, more homogeneous clusters in the test set, thereby increasing sequence similarity between the partitions and introducing similarity-driven bias into the results. Once the positive sequences were divided into training, validation, and test sets, we applied the same split to the negative sequences, ensuring that each negative sequence followed its corresponding positive sequence. This process resulted in a balanced dataset, maintaining a similar number of positive and negative samples in all sets. The dataset preparation pipeline is shown in Fig. 1A and B.

#### External validation dataset

To assess the generalizability of our models on a completely unseen species, we constructed an independent external test set from a ninth organism, *C. familiaris* (dog). Promoter sequences were collected from the EPDnew database [41](downloaded October 2025), based on the canFam3 genome assembly. The initial dataset contained 7,321 promoter sequences. Since this dataset was intended solely for testing and not training, we first removed sequences containing the ambiguous nucleotide ’N,’ resulting in 6540 sequences. To prevent data leakage, we applied CD-HIT clustering to compare the *C. familiaris* sequences against the combined training datasets of the eight organisms, utilizing a sequence identity threshold of 80%. We retained only those *C. familiaris* sequences that remained as single-member clusters (i.e. did not cluster with any training sequence). This filtering process yielded a final external test set of 6138 sequences distinct from the training data. Evolutionary divergence times (million years ago, MYA) between *C. familiaris* and the eight training organisms were obtained from the TimeTree database [45] using *Canis lupus familiaris* as the reference species.

### Models and tokenizers

We first tokenized the data by training four different tokenizers on our positive training set: the non-overlapping 6-mer, overlapping 6-mer, BPE, and WPC tokenizers. Each sequence was then converted into a matrix representation by embedding the tokens as numerical vectors for pretraining. We trained the BPE and WPC tokenizers using different stopping criteria to ensure a fair comparison: BPE with a fixed vocabulary size of 4250, and WPC with a frequency threshold that produced a vocabulary of approximately 4350 tokens. These sizes are comparable to the k-mer tokenizers, whose vocabularies are larger due to the high number of k-mer tokens that contain the ambiguous nucleotide ’N,’ which is excluded from frequency-based token merging. Specifically, the non-overlapping 6-mer tokenizer produced 4421 tokens, and the overlapping 6-mer produced 5332 tokens. It is also worth noting that although a canonical 6-mer vocabulary consists of $4^6 = 4096$ combinations, the k-mer tokenizers include additional tokens due to ambiguous nucleotides such as ’N’, which represents uncertain base identities in the sequence. The length distribution of tokens generated by BPE and WPC is shown in Supplementary Fig. S2.

For pretraining, we utilized the *RobertaForMaskedLM* model [46], a variant of the BERT model known as RoBERTa, optimized for efficiency. Like BERT, RoBERTa employs a transformer-based architecture with multi-head self-attention and fully connected layers. We trained the model using the Masked Language Modeling (MLM) [47] approach, where certain tokens within the sequences are masked, and the model is trained to predict these masked tokens. The pretraining process was conducted exclusively on the positive sequences from the eight organisms mentioned earlier. For fine-tuning, we employed the *RobertaForSequenceClassification* model, adapted to work with the pretrained *RobertaForMaskedLM*, leveraging its weights as the starting point. The fine-tuning phase aimed to train the model to classify sequences as promoters or non-promoters, using both their positive and negative sequences from the training dataset. All models for pretraining and fine-tuning were trained with a batch size of 8, using the *Adam* optimizer algorithm. A systematic hyperparameter search was performed for each model to ensure a fair comparison. The full details of this optimization process, including the search space and final values, are available in the Supplementary table S1.

### Explainable evaluation framework

We developed a position-wise SHAP importance analysis to identify specific regions within promoter sequences that contribute most to the model’s predictions. For each organism, we selected the top 100 positive test sequences with the highest predicted probabilities of being promoters. SHAP values were extracted for all tokens in the order they appear in the tokenized sequence, and token-level SHAP scores were then duplicated across the corresponding nucleotides to preserve positional continuity at the nucleotide level. We then averaged SHAP importance values across all selected sequences at each promoter position from 0 to 600 (corresponding to positions −300 to +300 relative to the TSS). This procedure allowed us to capture positional trends in model attention across the input sequences. To verify whether the observed patterns are unique to promoter sequences or artifacts of the negative data generation process, we repeated the analysis on the 100 negative sequences most confidently classified as non-promoters.

### Transfer learning for evolutionary-informed pretraining

Transfer learning is a widely used approach in machine learning for improving model performance in target domains with limited labeled data. In the context of genomics, evolutionary conservation between species suggests that regulatory patterns and sequence features may be shared across organisms, providing an opportunity to leverage data from related organisms [37, 38]. Building on this concept, we designed an evolutionary-informed pretraining strategy to enhance promoter classification performance. For this experiment, we used all available training data from the eight organisms, excluding 10,000 *H. sapiens* sequences (5000 promoter and 5000 non-promoter sequences) that were held out specifically for the fine-tuning stage. We compared two pretraining strategies: (i) a flat pretraining strategy, in which promoter sequences from all organisms are pooled without regard to evolutionary relatedness, and (ii) an evolutionary-informed pretraining strategy, which progressively focuses on species most closely related to the target organism.

The pretraining process consisted of 10 epochs. During the first five epochs, pretraining was performed using promoter sequences from all organisms in both the flat and evolutionary-informed settings. In the final five epochs, the evolutionary-informed model was trained using sequences from only the four species evolutionarily closest to *H. sapiens* (*M. mulatta, M. musculus, R. norvegicus*, and *H. sapiens* itself), according to the TimeTree resource [45]. In contrast, the flat model continued training on promoter sequences from all organisms, resulting in nearly twice as many training observations in this phase compared to the evolutionary-informed approach. After pretraining, both models were fine-tuned on the set of 10,000 held-out *H. sapiens* sequences. Model performance was then evaluated on the *H. sapiens* test set to assess the impact of the two pretraining strategies on promoter classification. To ensure that the observed trends were not dependent on a specific data partition, the entire experiment was repeated across five different training-validation splits, and the results were averaged.

## Results

### Evaluation of tokenization methods

First, we compared the performance of four different tokenization methods, as illustrated in Fig. 1C–E. This experiment is divided into three stages: pretraining, finetuning, and evaluation. During the **pretraining** stage, we trained four distinct BERT models, each using a different tokenization method: BPE, WPC, overlapping 6-mer, and non-overlapping 6-mer, using only the positive samples from the 65% training set across all organisms Fig. 1C. In overlapping k-mer tokenization, each token differs from the next one by just one nucleotide, creating dense and highly contextual representations. In contrast, non-overlapping k-mers divide the sequence into adjacent chunks without redundancy, resulting in a more compact but potentially less expressive representation. In comparison, BPE and WPC dynamically generate tokens of varying lengths based on frequency patterns in the training data, allowing them to capture both short and long motifs without being constrained to fixed-length segments.

**Figure 2.**
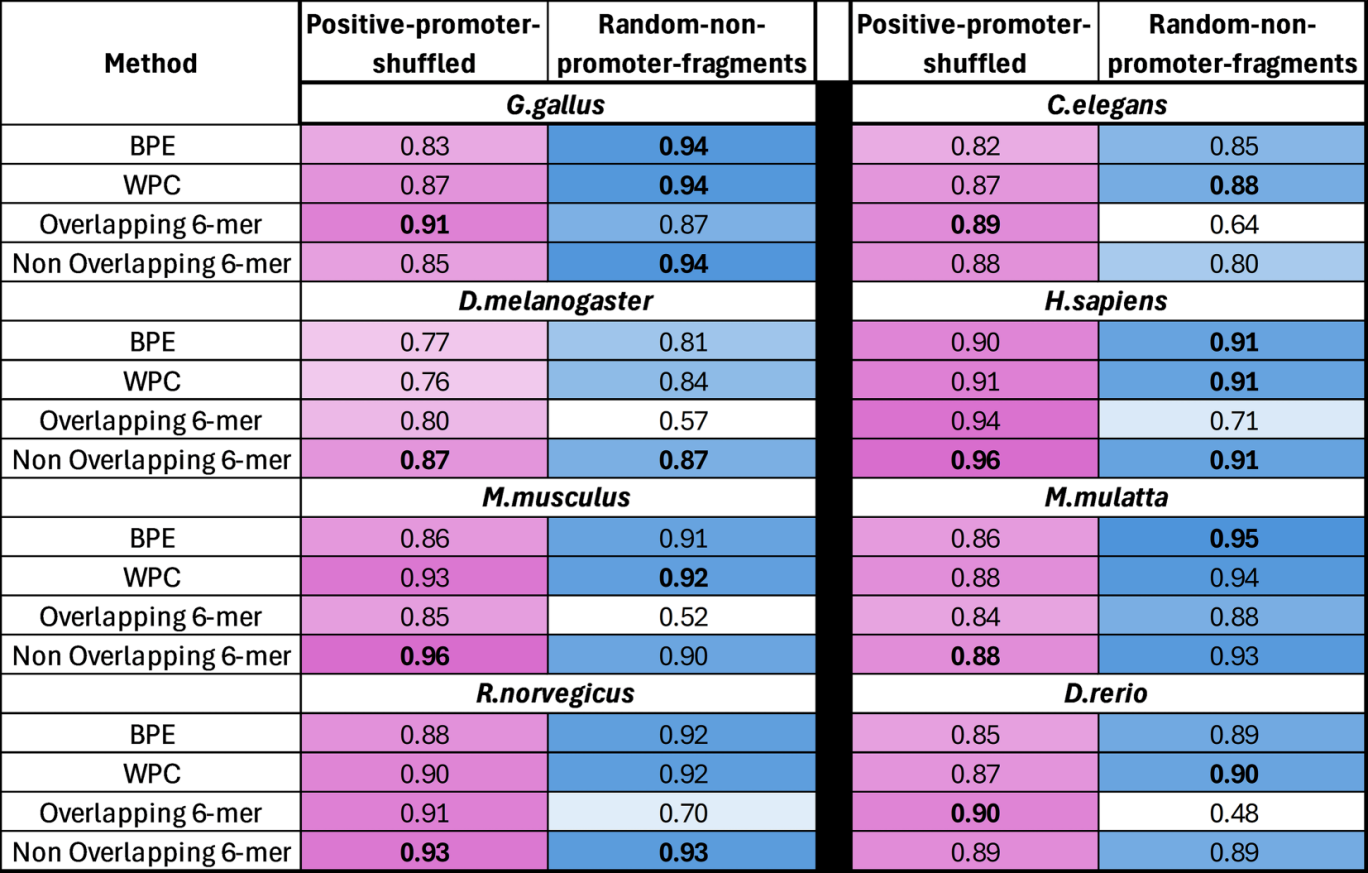
Performance comparison (AUC) of four tokenization methods (rows): BPE, WPC, overlapping 6-mer, and non-overlapping 6-mer, evaluated under two negative data generation strategies (columns): positive-promoter-shuffled or random-non-promoter-fragments, across eight organisms. The color gradient represents AUC values, with lighter colors indicating lower performance and darker colors indicating higher performance. The best-performing tokenizer for each organism:negative-set combination is indicated in bold font.

In the **finetuning** stage, each pretrained model was fine-tuned for a specific organism using both positive and negative samples from its corresponding 65% training and 15% validation sets (Fig. 1D), enabling the model to learn promoter sequence classification. This resulted in 32 fine-tuned models (8 organisms × 4 tokenization methods). Finally, in the **evaluation** stage, we assessed each model’s performance on its corresponding 20% test set (Fig. 1E) using the Area Under the Receiver Operating Characteristic Curve (AUC) metric. To ensure our findings reflect true model learning rather than biases introduced by the negative data generation strategy, we conducted the experiment twice using two negative datasets generated with different approaches: positive-promoter-shuffled and random-non-promoter-fragments (Fig. 2).

In most organisms, the non-overlapping 6-mer tokenization method outperformed the overlapping 6-mer approach. Specifically, when using the positive-promoter-shuffled negative data, the non-overlapping variant outperformed the overlapping variant in all organisms except *G. gallus, C. elegans*, and *D. rerio* (Fig. 2), three organisms with relatively small training sets (Table 1). When using the random-non-promoter-fragments negative data, the non-overlapping tokenizer achieved superior performance over the overlapping variant across all organisms without exception (Fig. 2).

The non-overlapping 6-mer tokenizer consistently outperformed both BPE and WPC across all organisms, except for *G. gallus*, where its performance was slightly lower when evaluated using the positive-promoter-shuffled negative data (Fig. 2). However, this advantage diminished under the random-non-promoter-fragments negative data; in this setting, BPE and WPC performed comparably or better in several species. Specifically, they outperformed the non-overlapping 6-mer in *C. elegans, M. mulatta, D. rerio*, and *M. musculus*. The first three species have smaller training sets, while *M. musculus* has one of the largest datasets. Although a shift in performance trend is observed between the two negative data strategies, *D. melanogaster* stands out as an example where the non-overlapping 6-mer outperformed both BPE and WPC in both negative data settings. The overlapping 6-mer showed improvement over BPE and WPC, in most species, except *M. musculus* and *M. mullata*, when using the positive-promoter-shuffled negative data; however, it performed much worse with random-non-promoter-fragments negative data.

To assess the statistical significance of performance differences between tokenization methods, we employed the DeLong test [48] on the AUC metric (Supplementary Fig. S3, Supplementary table S3.A), a standard evaluation approach in genomic sequence classification tasks [17, 19]. The resulting p-values were then adjusted for multiple testing using the Benjamini–Hochberg procedure to control the false discovery rate (FDR). This analysis allowed us to determine whether observed performance differences reflect consistent model advantages or are likely due to random variation. In all cases where the non-overlapping 6-mer tokenizer achieved a higher AUC than the overlapping 6-mer, the differences were statistically significant. When comparing the non-overlapping 6-mer tokenizer to BPE and WPC, under the positive-promoter-shuffled negative data method, where it generally outperformed both, the advantages were statistically significant, with particularly low p-values in organisms such as *M. musculus, D. melanogaster*, and *H. sapiens*. Under the random-non-promoter-fragments strategy, *D. melanogaster* again showed significant improvement with the non-overlapping 6-mer tokenizer, consistent with the trend observed in the positive-promoter-shuffled method. In contrast, for *C. elegans*, the statistically significant difference reflected an advantage of BPE and WPC over the non-overlapping 6-mer. Given the consistently high AUC values (ranging from 0.80 to 0.96) and the overall superior performance of the non-overlapping 6-mer tokenizer across most organisms, we selected it as the primary method for interpretability and transfer learning analyses, while retaining all tokenization methods for an external validation dataset to assess generalizability.

### SHAP-based positional importance analysis

**Figure 3.**
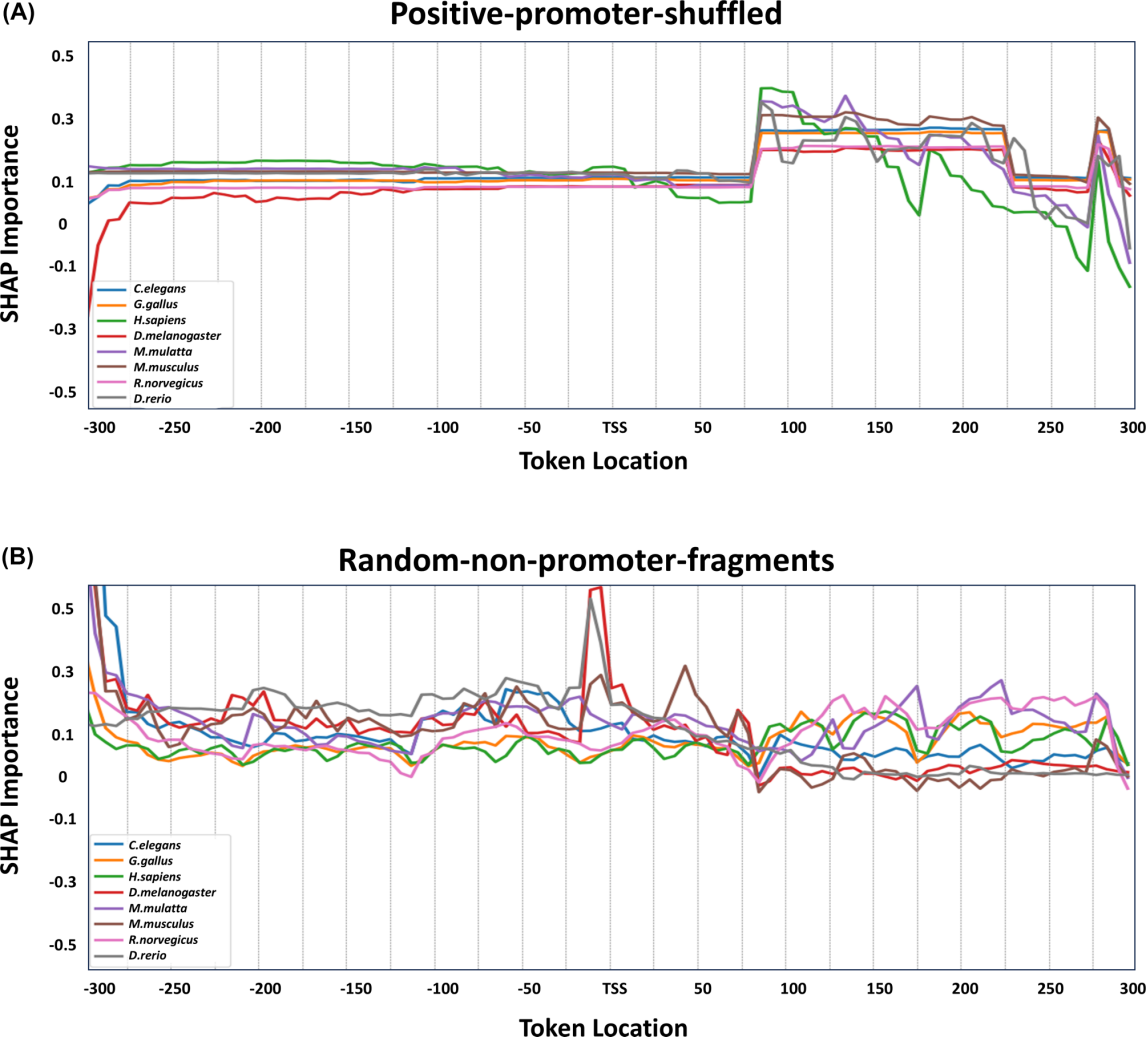
Positional SHAP analysis for promoter sequences. Shown are the average SHAP importance values (*y*-axis) across genomic positions (*x*-axis) relative to the TSS (−300 to +300 bp) for the top 100 **positive sequences** most confidently classified as promoters by the non-overlapping 6-mer model. Each curve represents a different organism (8 in total). SHAP values can be positive or negative, indicating whether each position increased or decreased the model’s confidence in the promoter classification. The results are shown for two negative data generation strategies: **(A)** positive-promoter-shuffled and **(B)** random-non-promoter-fragments.

To better understand which regions of promoter sequences influence model predictions, we applied position-wise SHAP importance analysis on the top 100 positive test sequences with the highest predicted probabilities of being promoters, selected separately for classifiers trained with different negative datasets. Using the positive-promoter-shuffled negative data, a distinct peak in SHAP importance appears around token location 390 (corresponding to +90 nucleotides downstream of the TSS), and is consistently observed across multiple organisms, most notably in *H. sapiens* (Fig. 3A). This positional trend is absent when using the random-non-promoter-fragments negative data (Fig. 3B), suggesting that the model may have learned to rely heavily on a specific positional feature only when trained using the positive-promoter-shuffled negative data.

To assess whether this effect is specific to positive sequences or an artifact of the negative data generation process, we repeated the same position-wise SHAP importance analysis on the 100 negative sequences most confidently classified as non-promoters. The resulting SHAP profiles (Supplementary Fig. S4) show no distinct peaks, especially in the positive-promoter-shuffled negative method, supporting that the observed positional pattern was learned from true promoter sequences, as no such pattern was detected in the examined negative sequences.

To further investigate this phenomenon, we focused on the *H. sapiens* dataset, where the effect was most evident, and analyzed the 100 positive test sequences with the highest predicted probabilities of being promoters. We compared the nucleotide distribution at position 390 to that at position 384 (+90 versus +84, Supplementary Fig. S5) to assess whether the observed SHAP peak could be attributed to an unusual nucleotide composition. The distributions appear highly similar, suggesting that the observed importance peak is unlikely to be driven by a simple compositional bias at that position.

Finally, to move beyond the averaged positional trends and investigate the specific sequence features the model learned, we examined the individual tokens with the highest SHAP values for the top five positive sequences in *H. sapiens* (Supplementary Fig. S6). This analysis revealed distinct patterns that differ between the two negative data strategies. In the positive-promoter-shuffled model (Supplementary Fig. S6A), the analysis identified a diverse set of tokens. This likely reflects the negative data generation process itself; the shuffled method, which preserves the original nucleotide composition, forces the model to rely on more mixed, motif-like features to distinguish promoters. Consequently, the features learned under this strategy provide more substantial evidence that the model is capturing a sequence-specific ’regulatory grammar.’ In contrast, in the random-non-promoter-fragments model (Supplementary Fig. S6B), the high-importance tokens show a prominent frequency of C and G nucleotides (e.g. ’GGCGGC’, ’GCCGCC’). This suggests that the model primarily relies on identifying localized CpG-rich signatures or short-range GC-enrichment to distinguish promoters from the genomic background.

### Evolutionary-informed pretraining

Next, we examined whether taking into account evolutionary relationships between organisms during pretraining can enhance model performance. To that end, we compared two distinct pretraining approaches: flat pretraining and evolutionary-informed pretraining, both utilizing the non-overlapping 6-mer tokenization, which showed superior performance in our tokenizer comparison.

In the flat pretraining approach, we simultaneously used promoter sequences from all organisms throughout all pretraining epochs. In contrast, the evolutionary-informed pretraining approach involved using promoter sequences from all organisms for the first half of the pertaining epochs, followed by sequences only from the four species evolutionarily closest to *H. sapiens* (*M. mulatta, M. musculus, R. norvegicus*, and *H. sapiens*), as determined using the TimeTree resource [45]. Both models were evaluated on the *H. sapiens* test set using AUC as the performance metric. We fine-tuned a series of *H. sapiens-*specific models, differing only in the amount of *H. sapiens* training data used. Starting with 1000 sequences, we incrementally added 1000 additional sequences at each fine-tuning step, up to a total of 10 000 sequences.

**Figure 4.**
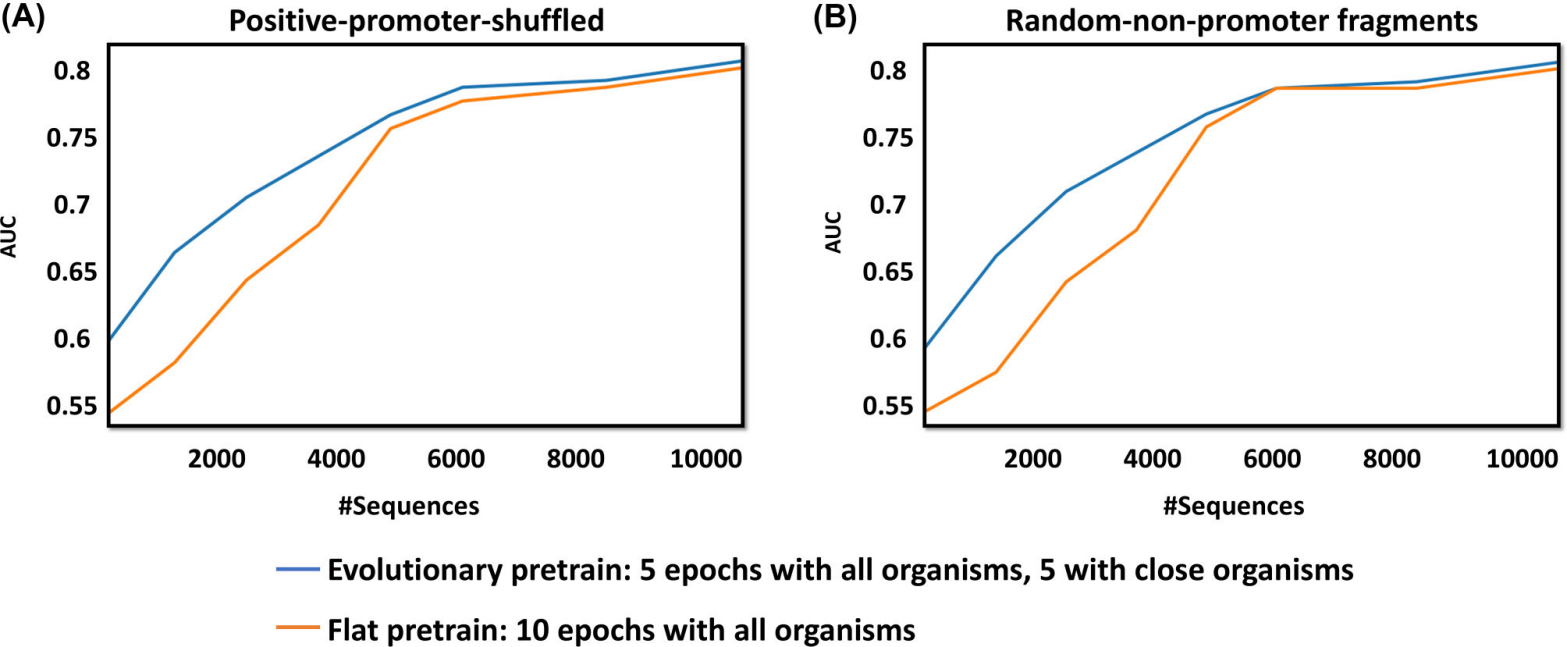
Evaluating the advantage of evolutionary-informed pretraining. The figure compares AUC scores of models pretrained using two strategies: evolutionary-informed pretraining and flat pretraining, across increasing amounts of *H. sapiens* fine-tuning data, ranging from 1000 to 10 000 sequences (equal amounts of promoter and non-promoter sequences), in increments of 1000. In the evolutionary-informed strategy, pretraining was performed using promoter training data from all eight organisms for the first five epochs, followed by promoter training data from only the four species evolutionarily closest to *H. sapiens* during the final five epochs. In the flat strategy, promoter training data from all eight organisms were used throughout all ten pretraining epochs. Fine-tuning was conducted on the 10 000 held-out *H. sapiens* sequences, and model evaluation was performed on the *H. sapiens* test set. The results are shown for two types of negative datasets used during training: **(A)** positive-promoter-shuffled and **(B)** random-non-promoter-fragments.

The performance of the two pretraining strategies is shown in Fig. 4, using both negative data generation methods: positive-promoter-shuffled (Fig. 4A) and random-non-promoter-fragments (Fig. 4B). In both methods, the evolutionary-informed pretraining consistently outperformed flat pretraining when using up to 5000 fine-tuning sequences. This indicates that incorporating sequences from evolutionarily close organisms during pretraining provides a clear advantage when labeled data are limited. However, as the fine-tuning set grew beyond 5000 sequences, the performance gap narrowed in both cases, suggesting that the choice of pretraining strategy becomes less critical when sufficient task-specific data are available.

### External validation on an unseen organism

**Figure 5.**
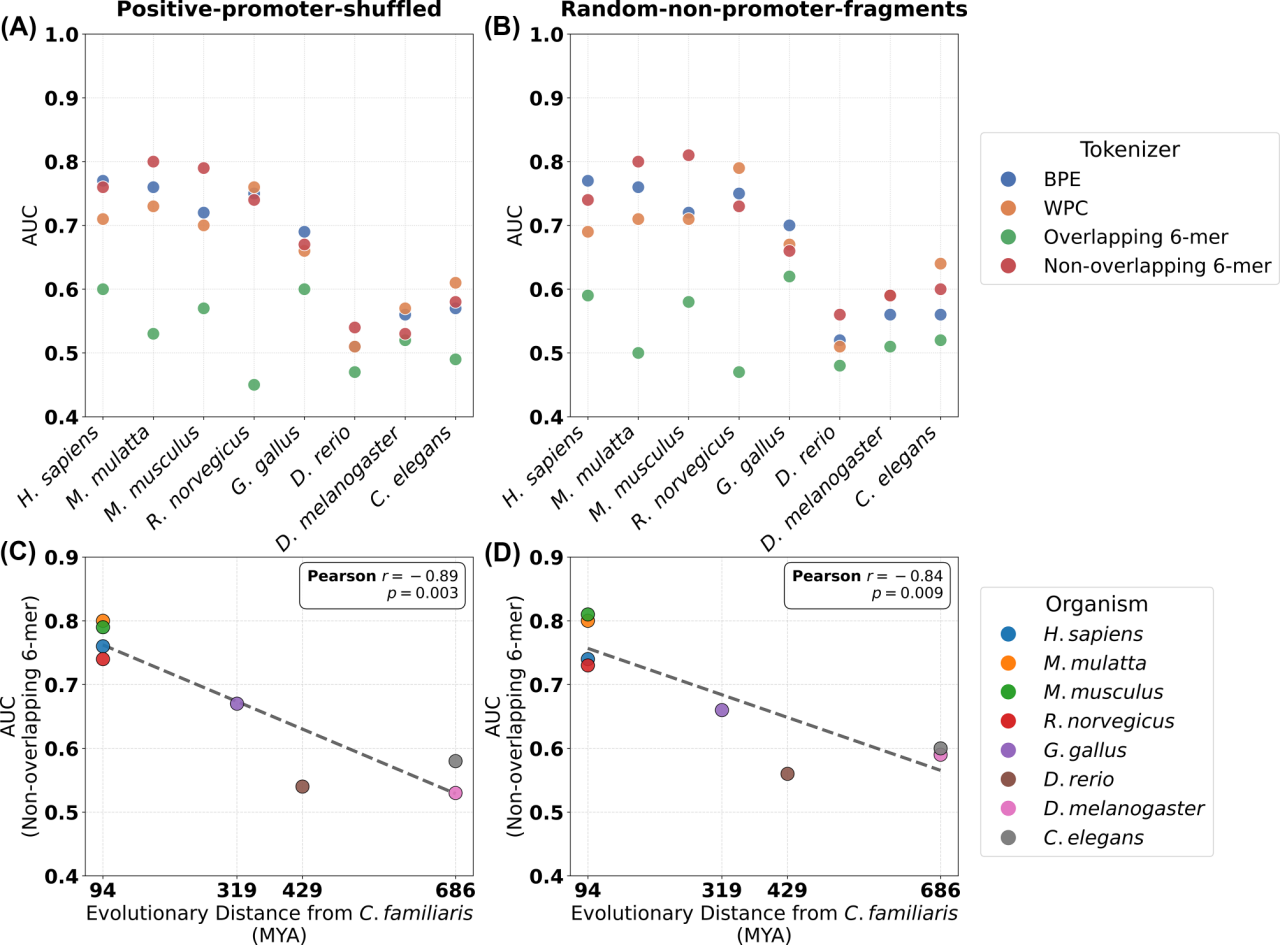
External validation of model generalizability on an unseen organism, *C. familiaris*. The figure displays the performance (AUC) of models trained on eight different organisms (listed in Table 1) and evaluated on an independent, external test set from a ninth organism, *C. familiaris* (dog). **(A** and **B)** Performance comparison of four tokenization methods. The x-axis denotes the organism used for training, ordered by evolutionary distance from *C. familiaris*, and point colors indicate the tokenization method. **(C** and **D)** Relationship between evolutionary distance and model performance for the non-overlapping 6-mer tokenizer. The *x*-axis denotes the evolutionary distance between the training organism and *C. familiaris* (in million years ago, MYA), and point colors indicate the training organism. Dashed lines represent linear regression fits, with the Pearson correlation coefficient ($r$) and corresponding *P*-value displayed in each panel. Results are shown for two negative data generation strategies: (**A** and **C**) positive-promoter-shuffled and (**B** and **D**) random-non-promoter-fragments.

To assess the generalizability of our models beyond the organisms included in training, we evaluated them on a new, unseen external test set from *C. familiaris* (described in the 'External validation dataset' Section). Across these cross-species evaluations, models employing the non-overlapping 6-mer tokenizer maintained robust and generally superior performance, reinforcing its effectiveness even under the more challenging external-validation scenario (Fig. 5A and B).

Focusing specifically on the non-overlapping 6-mer tokenizer, we observed that model performance exhibits a strong negative correlation with the evolutionary distance between the training organism and *C. familiaris* (Fig. 5C and D). Notably, performance remained relatively high for the four species evolutionarily closest to *C. familiaris* (*H. sapiens* to *R. norvegicus*), forming a distinct high-performance cluster, followed by a pronounced decline for more distantly related organisms. This trend was supported by a Pearson correlation coefficient of $r = -0.89$ ($p = 0.003$) under the positive-promoter-shuffled strategy (Fig. 5C) and $r = -0.84$ ($p = 0.009$) under the random-non-promoter-fragments strategy (Fig. 5D). These results indicate that evolutionary proximity between training and testing species is a strong predictor of cross-species generalization performance.

## Discussion

Our study addresses key challenges in applying large language models (LLMs) to genomic sequence classification, specifically in the context of promoter detection across multiple species. We investigated how different tokenization schemes, evaluation setups, and training data constraints affect model performance and interpretability. Our findings contribute to a growing body of work focused on adapting modern NLP techniques to biological sequence data, offering practical insights for future modeling strategies.

### Effects of negative data generation methods on classification performance

The strategy for generating negative training data plays a critical role in shaping the performance and interpretability of genomic classifiers. While most prior studies rely on a single negative sampling approach, we compared two commonly used methods: the positive-promoter-shuffled method, which substitutes parts of real promoter sequences to create biologically plausible negatives [18, 35], and the random-non-promoter-fragments method, which samples arbitrary genomic regions far from TSS [19].

Our comparison revealed that the non-overlapping 6-mer tokenizer consistently outperformed or matched the performance of the overlapping 6-mer tokenizer across both negative data generation strategies. However, the advantage of the non-overlapping 6-mer tokenizer was less consistent when compared to subword-based methods, BPE and WPC. When using the positive-promoter-shuffled negative data, the non-overlapping 6-mer tokenizer demonstrated a clear and statistically significant performance advantage over both subword methods (BPE and WPC). In contrast, under the random-non-promoter-fragments method, this performance gap narrowed substantially, with BPE and WPC performing comparably, or slightly better, in several organisms, although a statistically significant difference in their favor was observed only in *C. elegans*.

This shift likely reflects differences in task difficulty posed by the two strategies. The shuffled method preserves key promoter features (e.g. TATA-box, TSS proximity), enabling the non-overlapping 6-mer tokenizer to leverage precise and localized motifs. In contrast, the random fragment method introduces highly variable and unstructured negatives. The non-overlapping tokenizer, which segments sequences into discrete, fixed-length tokens, may struggle in such noisy contexts where clear biological patterns are sparse or absent. Subword tokenizers, with their flexible tokens, may generalize better in these conditions. The pronounced performance drop in *C. elegans*, a species evolutionarily distant from the others, may indicate that the composition of distal intergenic regions used as negatives in this method varies more drastically across organisms, complicating learning.

Our SHAP-based positional analysis further underscores the influence of negative data generation on model behavior. The presence or absence of positional importance signals, such as the distinct peak downstream of the TSS, depended on the negative sampling method. Taken together, these findings suggest that the choice of negative data generation strategy not only affects performance but also determines which features the model learns to rely on. Consequently, training on shuffled negatives may be particularly advantageous for future generative applications, where capturing the underlying syntax, rather than global compositional statistics, is essential for designing synthetic regulatory elements. As such, tokenizer selection should be aligned with the structural characteristics of the negative data. A possible future direction would be to systematically evaluate the performance of tokenizers under varying degrees of perturbation to positive sequences, providing clearer guidelines for designing biologically realistic and effective training datasets.

### Methodological considerations and alternative tokenization approaches

Our study focused on tokenization methods capable of capturing multi-character biological motifs. For completeness, we also evaluated a character-level tokenizer (Supplementary Fig. S7, Supplementary Table S3B). Its performance was significantly lower (FDR < 0.05) than the assessed tokenizers in our primary analysis (Fig. 2), which use tokens capable of representing motifs. Specifically, the character-level performance was consistently inferior to that of the maximum (max) performing multi-character tokenizer. This aligns with our hypothesis that motif-level information is advantageous for this specific problem. While prominent models like HyenaDNA [25] and Caduceus [26] successfully use character-level inputs, they rely on architectures fundamentally different from the standard attention-based LLMs investigated here. Moreover, the character-level tokenizer only outperformed the minimum (min) performing multi-character tokenizer (overlapping 6-mer) in the random-non-promoter-fragments negative data strategy, and mostly when the min tokenizer showed near-random performance. This outcome, which highlights a critical sensitivity of the overlapping k-mer approach to contextual noise, is more indicative of the overlapping tokenizer’s failure than the character tokenizer’s success. Furthermore, the evaluations of these tools often do not apply sequence-identity filtering tools such as CD-HIT, which are essential for preventing data leakage between training and testing sets. This limits the direct comparability of their reported performance to ours. Overall, these observations suggest that the optimal tokenization strategy is highly dependent on both the model architecture and the rigor of the data splitting methodology.

### Evidence for positional signals near the TSS

To determine whether the models capture biologically meaningful positional signals within promoter sequences, we analyzed the distribution of SHAP importance scores relative to the TSS. Specifically, in models trained using the positive-promoter-shuffled negative generation method, we observed a prominent SHAP importance peak around +90 nucleotides downstream of the TSS. This positional trend was notably absent in the sequences classified as non-promoters with high confidence (Supplementary Fig. S4), reinforcing that the learned pattern is promoter-specific rather than a general sequence artifact. To further investigate the origin of this signal, we compared the nucleotide distribution at the peak position to that of adjacent, low-importance positions (Supplementary Fig. S5). The distributions were highly similar, ruling out the possibility that the model merely exploited a simple compositional bias or a specific nucleotide artifact at that location. Collectively, these findings suggest that the model has learned to leverage biologically plausible positional information as part of its decision-making process, rather than relying solely on sequence composition or dataset-specific artifacts.

### Possible effect of ambiguous tokens

During our analysis, we observed that the vocabularies generated by both k-mer tokenizers contained many tokens with ambiguous nucleotides, particularly ‘N.’ In contrast, BPE and WPC, which rely on frequency-based vocabulary construction, tend to exclude such tokens due to their low occurrence. As a result, BPE and WPC vocabularies are more constrained and may lack representations for uncertain or rare sequence motifs. To ensure that the model was not simply learning to distinguish between positive and negative samples based on the distribution of ’N’s, we compared their frequencies, confirming their extreme sparsity in both the positive sequences and the random-non-promoter-fragments negative set (Supplementary Table S2). For the positive-promoter-shuffled negative set (not shown in the table), the nucleotide counts are identical to the positive set by definition, as this set was generated by shuffling the positive sequences. Therefore, the distribution of ’N’s is not a significant confounding variable in either negative data strategy.

Although ambiguous nucleotides such as ‘N’ are rare in high-quality curated datasets like EPDnew (Supplementary Table S2), the way they are handled highlights a key distinction between tokenization strategies. Frequency-based methods such as BPE and WPC effectively exclude ‘N’-containing tokens from the learned vocabulary due to their scarcity in the training data. In contrast, k-mer tokenization relies on a fixed, exhaustive vocabulary that naturally includes combinations containing ‘N.’ Even if these tokens are rarely encountered during training on positive sequences, their inclusion helps to reduce overfitting.

### Validation of the pretraining strategy

Using a pretrained model is a standard approach in the field, with foundational models like DNABERT (pretrained on the human genome) serving as a common starting point. This strategy is particularly effective for data-rich organisms like *H. sapiens*, which dominate many promoter prediction studies. In contrast, our framework includes eight organisms with smaller genomes and more limited promoter-annotated datasets, which poses a challenge for LLMs.

We follow a parallel principle: since this study’s central goal was to systematically compare tokenization methods, we performed the pretraining phase exclusively on our positive sequences to ensure a consistent framework. This approach ensures the model learns from ‘real,’ experimentally verified data (our positive set), rather than from our artificially generated negative sequences, which could introduce artifacts. While our current strategy focuses on adapting the model to our specific domain, future work could also explore pretraining on different, additional genomic tasks to assess potential knowledge transfer.

To assess the value of this pretraining strategy, we included an additional baseline in which the pretraining phase was omitted. In this baseline, models were trained directly on the fine-tuning data (the balanced positive and negative sets for each organism). The results, comparing the ‘no pretrain’ and ‘with pretrain’ strategies, demonstrate that while our positive-only pretraining strategy provides a statistically significant performance boost (Supplementary Fig. S8, Supplementary Table S3C), the overall trends are preserved. Specifically, the relative performance advantages of the tokenizers, as observed in our main analysis (Fig. 2), remain consistent.

### The impact of promoter length on model performance

Our initial choice of a 601 bp window (−300 to +300) was a deliberate decision to create a comprehensive model capable of capturing a wide range of potential regulatory elements, encompassing the different windows used in various established studies. However, we acknowledge that input sequence length is a critical parameter that defines the context available to the model. To systematically evaluate the impact of this parameter, we conducted an additional experiment using a shorter, more standard promoter window of −250 to +50 bp. The results of this analysis (Supplementary Fig. S9, Supplementary Table S3D) revealed an interesting trend relative to our primary 601 bp analysis (Fig. 2). While most of the performance trends were preserved across tokenizers, the observed variations in AUC scores were not statistically significant in the majority of cases. Although the overlapping 6-mer tokenizer showed an upward trend on the random-non-promoter-fragments dataset, the overall lack of statistical significance prevents a definitive conclusion regarding the impact of the input window size. Consequently, we cannot unequivocally determine that the larger 601 bp window presents destructive sequence information or contextual noise.

### The impact of negative data perturbation on tokenizer performance

Our results, which favor k-mer tokenization, contrast with the reported success of subword-based methods in prominent models like GROVER [16] and the newer DNABERT 2 [49]. After our supplementary analysis ruled out promoter length as the cause, we identified the negative data generation strategy as the key differentiating factor. The methodologies are substantially different: DNABERT 2 creates its negative set using a hybrid approach of random genomic sampling and sequence substitution. Meanwhile, GROVER uses a sequence substitution similar to ours, but with a much higher shuffling perturbation rate (75%) than ours (32%). We conclude that the choice of negative data generation strategy alters the underlying patterns required for classification. Therefore, the optimal tokenizer is not universal but might depend on the negative sampling strategy.

### The effect of evolutionary distance on transfer learning and model generalization

Our experiments investigated evolutionary-informed pretraining and revealed a clear relationship between evolutionary distance and model performance. While the principle that pretraining on evolutionarily closer species improves performance is intuitive, our contribution lies in the quantitative demonstration of this effect for genomic LLMs and in identifying its practical limits under data-scarce conditions. Related studies, such as the Nucleotide Transformer [40], have also leveraged cross-species data, but have not systematically examined how evolutionary distance or data scale influence performance. We observed that pretraining on sequences from closely related organisms consistently produced superior performance, especially when fine-tuning was performed with fewer than 5000 observations. This finding provides a practical benchmark for genomic modeling in low-data regimes, suggesting that an evolutionary-informed pretraining strategy is highly effective for organisms with limited experimental annotations. However, for well-annotated organisms with larger training sets, the benefit of evolutionary-informed pretraining diminishes, and broader, less selective pretraining appears sufficient.

Interestingly, flat pretraining, despite using a larger number of training observations in the second half of the pretraining, actually performed worse. This suggests that excluding distant species during later pretraining stages can be beneficial, implying that training on more data is not necessarily advantageous when that data is biologically heterogeneous. This conclusion is supported by average performance across five different training-validation splits. Together, these results support the notion that closely related organisms share a more similar ‘DNA language’ than evolutionarily distant species. Since this ‘language’ is encoded in nucleotide sequences and their distributions, our findings suggest notable biological differences in promoter sequences between closely related organisms and those farther apart on the evolutionary scale.

Independent support for this conclusion comes from our external validation experiment ('External validation on an unseen organism' Section), where models trained on evolutionarily closer species generalized substantially better to the unseen organism *C. familiaris*. This demonstrates that the performance benefits observed in our transfer learning experiment (Fig. 4) are not only an artifact of the pretraining strategy but reflect genuine, shared biological patterns among related species, supporting our hypothesis of a shared ’DNA language.’ A recent study [50] proposed an alternative approach for weighting observations in training by appending RNA sequence labels as additional tokens to the end of tokenized RNA sequences. This method enables the model to prioritize sequences belonging to the same RNA type, a concept analogous to our evolutionary-aware pretraining and further emphasizing the value of custom token weighting strategies in genomic modeling.

### Key findings and future research directions

Our findings highlight several key methodological considerations. First, we found that input sequence may affect tokenizer efficacy; for example, the overlapping 6-mer tokenizer, which struggled on longer 601 bp sequences, showed significantly improved performance on shorter 300 bp windows, in some settings. Second, our pretraining strategy, which utilizes positive sequences, demonstrated a consistent performance boost compared to models trained without pretraining. This concept of data leveraging was further validated in our cross-species analysis, which strongly supports an evolutionary-informed data transfer approach. Models trained on species phylogenetically close to the target, as demonstrated in our external validation on *C. familiaris* (dog), generalized well. In contrast, models trained on distant organisms, such as *C. elegans*, failed. As a future direction, this research could be extended from data transfer to task transfer. We propose exploring whether pretraining on a different but related genomic task, such as enhancer identification, could provide valuable knowledge transfer to improve performance on the downstream task of promoter classification.

## Conclusions

Our study comprehensively evaluates LLM-based approaches for genomic promoter classification, focusing on tokenization strategies, model interpretability, and cross-species data utilization. We found that the non-overlapping 6-mer tokenizer consistently outperforms BPE, WPC, and overlapping 6-mer, particularly in organisms with sufficient training data. For organisms with limited training data, we demonstrate that evolutionary-informed transfer learning can significantly improve model performance by leveraging promoter sequences from closely related organisms. Additionally, our SHAP-based positional analysis revealed a distinct positional signal, which appears to be affected by the negative data generation method, highlighting the importance of the negative data generation method. While our evaluation focuses on a single downstream task, the promoter classification task, we underline that genomic LLMs are designed to support a broad range of genomic analyses. Future studies may expand the evaluation to additional tasks to assess the utility of different tokenization strategies. Together, these findings contribute to the ongoing development of LLMs in bioinformatics by highlighting the importance of tokenization techniques, pretraining strategies, and evaluation methods that are aligned with the unique characteristics of genomic data.

## Supplementary Material

lqag025_Supplemental_Files

## Data Availability

The positive and negative datasets, and the SHAP values, generated in this study, can be found at zenodo.org/records/18188117. The code is available at https://github.com/IsanaVekslerLublinsky/PromoterPrediction_Tokenizers and https://doi.org/10.6084/m9.figshare.29605871. To facilitate the replication of our results, the GitHub repository includes a Conda environment file (PromoterPrediction.yml) that specifies all necessary software packages and their exact versions, along with a detailed README.md file that provides step-by-step instructions for setting up the environment and executing the scripts.
