# Supplementary material for: Optimizing genomic language models for promoter prediction: a comparative study of tokenization and cross-species learning
Source: NAR Genom Bioinform. 2026 Mar 12;8(1):lqag025. doi: 10.1093/nargab/lqag025 (PMC12980338; doi:10.1093/nargab/lqag025)
Supplement: lqag025_Supplemental_Files [file lqag025_supplemental_files.zip › Suppl.pdf]

Optimizing genomic language models for promoter prediction: A comparative study of tokenization and cross-species learning

Eyal Hadad, Noia Kogman, Lina Golan, Anva Avraham, Reut Ben Hamo,
Zhi Wei, Lior Rokach, and Isana Veksler-Lublinsky

1 Hyperparameter optimization

For our promoter prediction task, we employed the RoBERTa architecture, which is highly effective for sequence classification. Given the relatively small dataset, we selected the RoBERTa-base model. This configuration balances performance and computational demand, featuring 12 layers, a hidden size of 768, 12 attention heads, and approximately 125 million parameters. After establishing this base model, we independently performed a systematic hyperparameter search for each of the four tokenization methods to ensure a fair and robust comparison. Optimization was conducted using a grid search on a dedicated validation set (15% of the data), targeting key hyperparameters that significantly impact model performance. The complete search space and the final optimal values selected for each method are detailed in Table S1. This process ensures that each tokenization-specific model was evaluated at its optimal configuration.

Table S1: Hyperparameter search space and optimal values for fine-tuning

**Hyperparameter**	**Tokenization method**	**Search space**	**Optimal value chosen**
**Learning rate**	Non-overlapping 6-mer	[1e-5, 3e-5, 5e-5]	3e-5
	Overlapping 6-mer	[1e-5, 3e-5, 5e-5]	3e-5
	BPE	[1e-5, 3e-5, 5e-5]	5e-5
	WPC	[1e-5, 3e-5, 5e-5]	5e-5
**Batch size**	Non-overlapping 6-mer	[8, 16, 32]	8
	Overlapping 6-mer	[8, 16, 32]	8
	BPE	[8, 16, 32]	8
	WPC	[8, 16, 32]	8
**Number of epochs**	Non-overlapping 6-mer	[1-15]	10
	Overlapping 6-mer	[1-15]	10
	BPE	[1-15]	10
	WPC	[1-15]	10

2 Sequence composition analysis

Table S2: Statistics on 'N' nucleotide content in the sequence data. Each row presents data for a specific organism, showing the total number of nucleotides. The subsequent columns detail 'N' statistics for positive (Pos) and negative (Neg) sequences (from random-non-promoter fragments method), including the percentage (%) and the absolute count (#).

**Organism**	**#Nucleotides**	**Pos %N**	**Pos #N**	**Neg %N**	**Neg #N**
H.sapiens	17,788,398	0.00%	1	0.00%	0
M.mulatta	5,007,532	0.08%	4,141	0.02%	1,142
M.musculus	15,091,110	0.00%	682	0.00%	0
R.norvegicus	7,573,201	0.13%	9,745	0.01%	767
G.gallus	3,681,726	0.01%	506	0.00%	0
D.melanogaster	10,198,970	0.00%	0	0.00%	200
D.rerio	6,447,528	0.03%	1,739	0.01%	883
C.elegans	4,279,120	0.00%	0	0.00%	0

3 Statistical significance data

Table S3: Comprehensive statistical significance test results. This dataset is provided as an external Excel file containing detailed statistical significance calculations based on the DeLong test, including raw and adjusted p-values using both Benjamini-Hochberg (FDR) and Bonferroni correction methods. The file comprises four tabs ("S3.A Tokenizers", "S3.B Character", "S3.C Pretrain", and "S3.D Length") that provide the raw statistical data supporting the significance annotations (white asterisks) shown in Figures S3, S7, S8, and S9, respectively.

4 Supplementary Figures

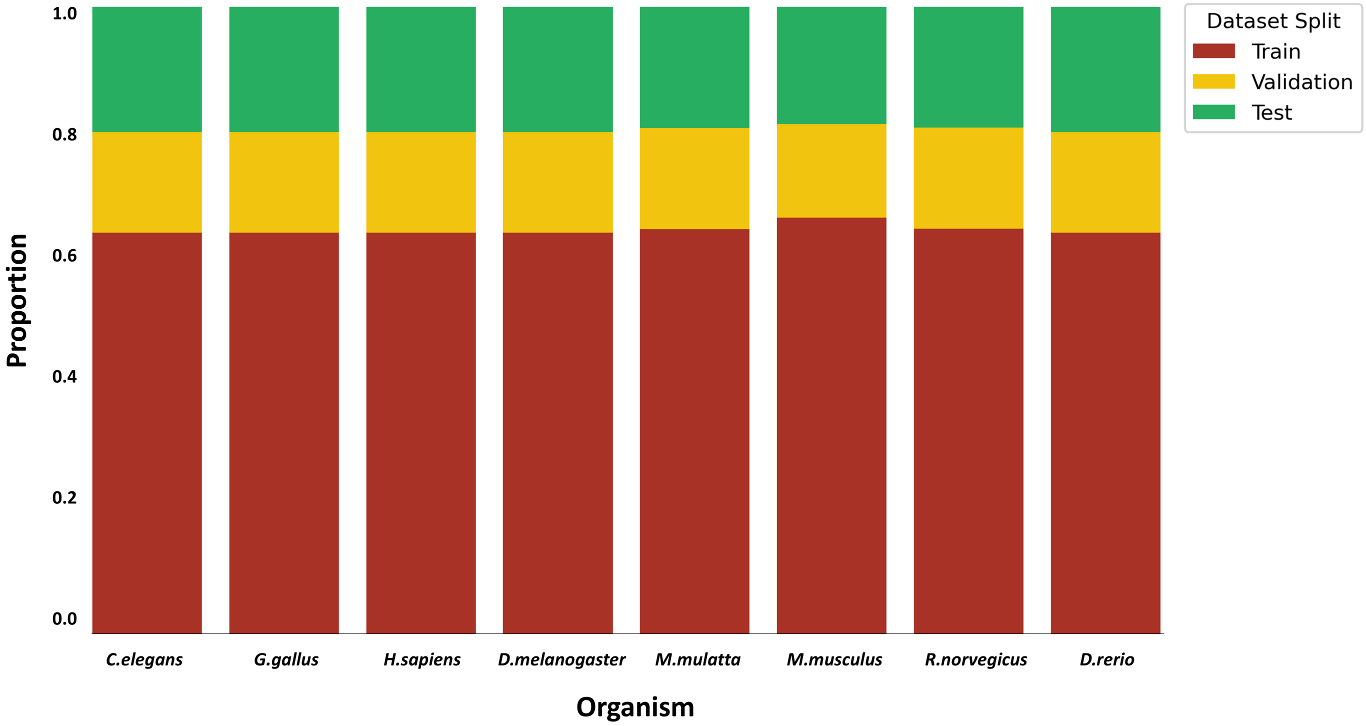

Fig. S1: Proportional distribution of training, validation, and test sequences within each organism. Each bar represents a single organism and is divided into three stacked segments corresponding to the dataset splits: red for training, yellow for validation, and green for test. The y-axis indicates the relative proportion within each organism (summing to 100%), and the x-axis lists the organisms.

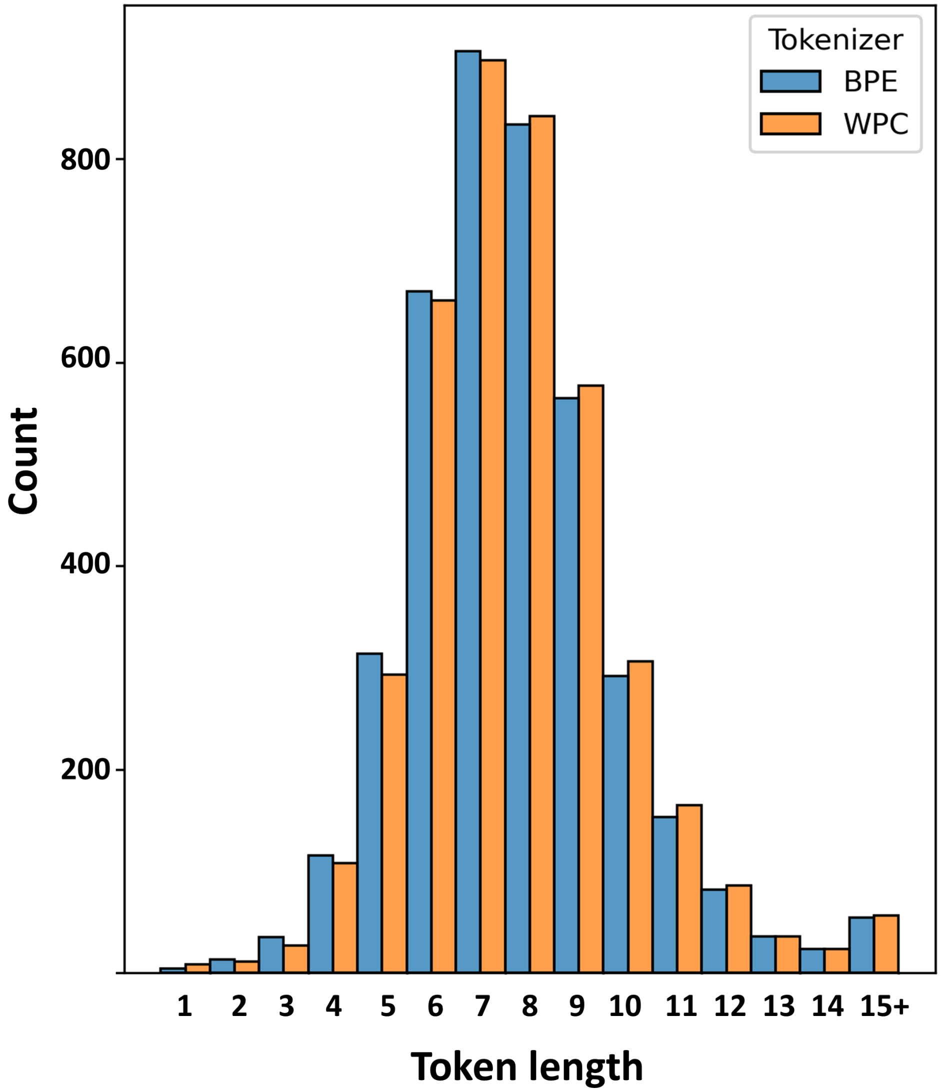

Fig. S2: Token length distribution for BPE and WPC tokenizers. The x-axis represents token length, ranging from 1 to 15+, where the final bin (15+) includes all tokens with a length of 15 or greater. The y-axis shows the count of the tokens. The blue bars correspond to tokens generated by the BPE tokenizer, while the orange bars represent tokens generated by the WPC tokenizer.

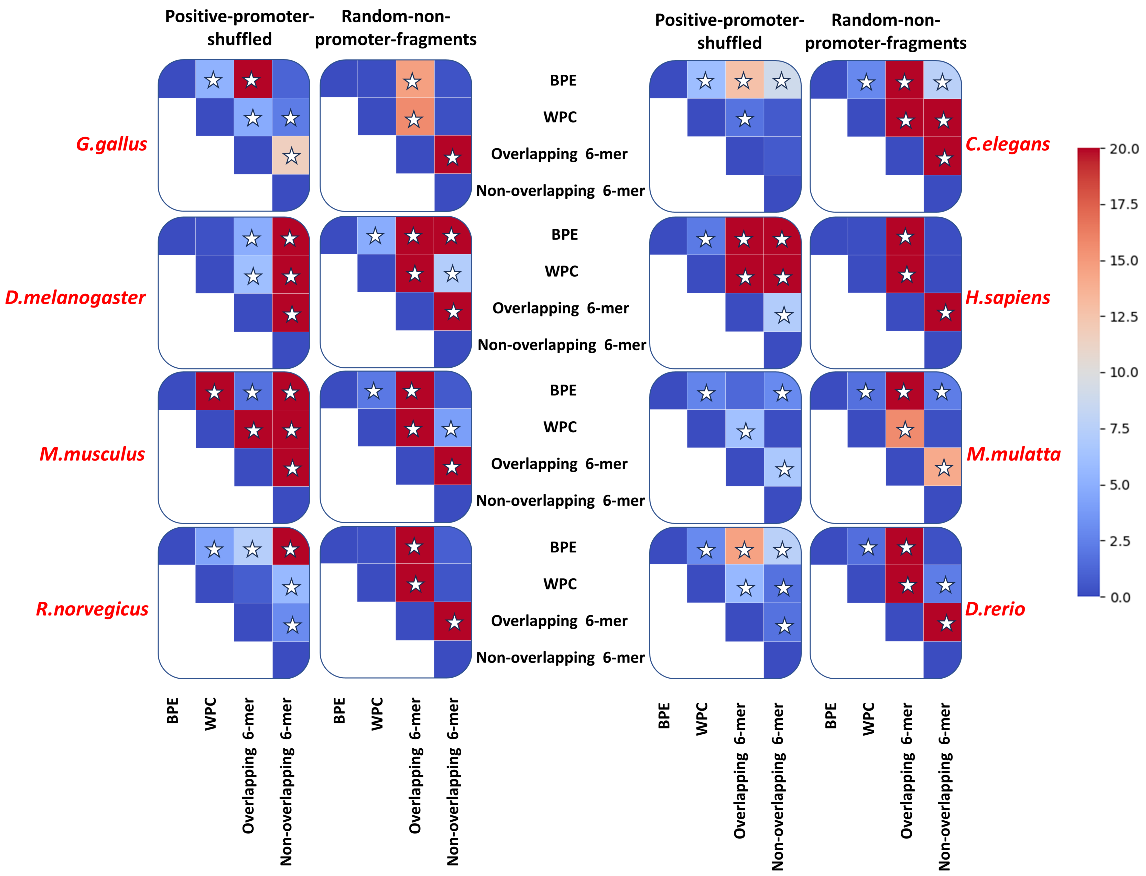

Fig. S3: Pairwise DeLong test results comparing AUC scores between tokenization methods across organisms and negative data generation strategies. Each subplot represents a different organism, with the left and right columns corresponding to the two negative data generation strategies: positive-promoter-shuffled and random-non-promoter-fragments. Within each matrix, rows and columns correspond to the tokenization methods: BPE, WPC, overlapping 6-mer, and non-overlapping 6-mer. The color gradient indicates the -$\log_{10}$(p-value) from the DeLong test, with darker red signifying stronger statistical significance. Dark blue cells denote comparisons with non-significant differences (unadjusted p-value > 0.05). Statistically significant comparisons after multiple testing correction (FDR < 0.05, Benjamini-Hochberg corrected) are marked with a white asterisk (*). The complete set of raw and adjusted p-values is provided in Table S3.A.

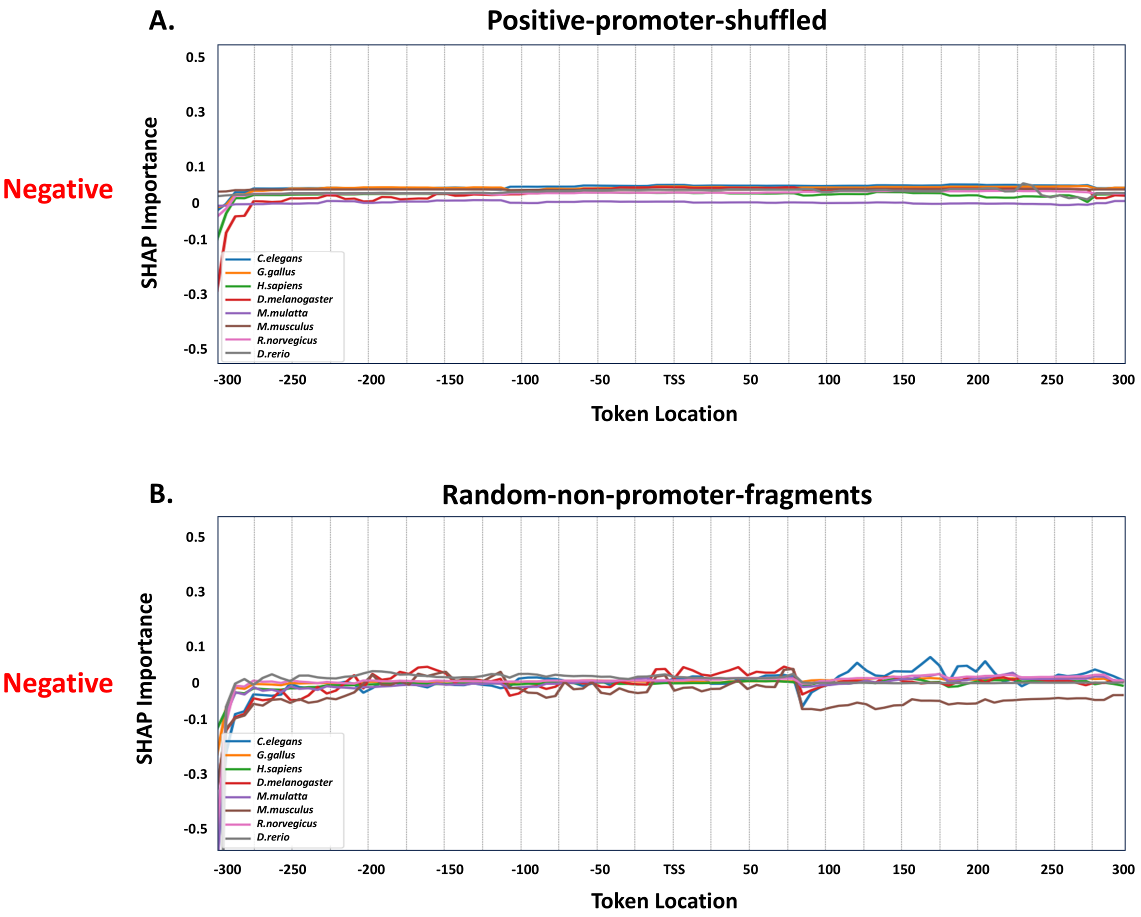

Fig. S4: Positional SHAP analysis for negative sequences. Shown are the average SHAP importance values (y-axis) across genomic positions (x-axis) relative to the TSS (-300 to +300 bp) for the 100 **negative sequences** most confidently classified as non-promoters by the non-overlapping 6-mer model. Each curve represents a different organism (8 in total). SHAP values can be positive or negative, indicating whether each position increased or decreased the model's confidence in the promoter classification. The results are shown for two types of negative datasets used during training: **A.** positive-promoter-shuffled and **B.** random-non-promoter-fragments.

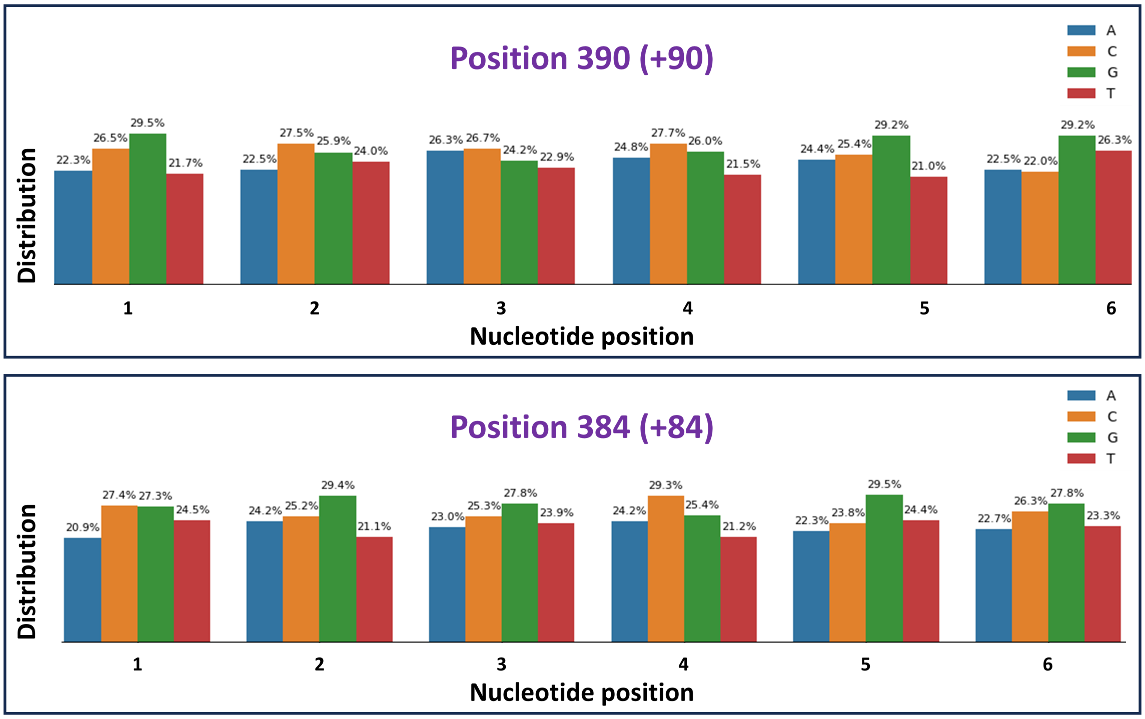

Fig. S5: Nucleotide composition at positions around peak SHAP importance corresponding to Figure 3.A. Shown are bar plots of the nucleotide frequency distribution (A, C, G, T) across the six positions of 6-mer tokens starting at positions +84 (bottom) and +90 (top) relative to the TSS. The analysis is based on the same 100 positive promoter sequences from the *H.sapiens* test set that were classified with the highest confidence as promoters by the non-overlapping 6-mer model trained using the positive-promoter-shuffled negative strategy. Each color represents a nucleotide, as indicated in the legend. The percentages represent the nucleotide composition at each specific position of the 6-mer and sum to 100%.

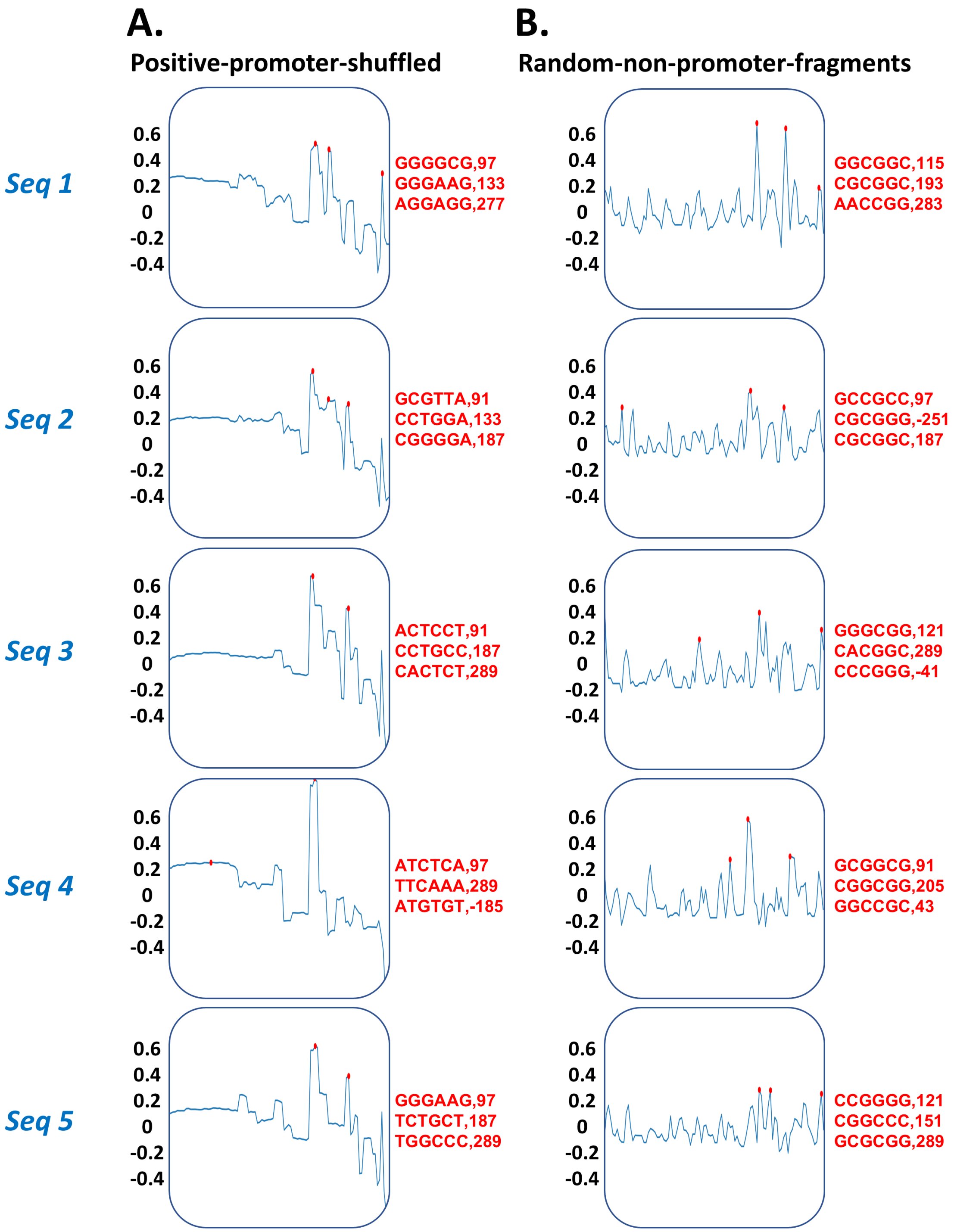

Fig. S6: Individual SHAP importance profiles for the top 5 *H. sapiens* promoter sequences. Shown are SHAP importance plots for the five *H.sapiens* positive test sequences most confidently classified as promoters by the non-overlapping 6-mer model (corresponding to Figure 3). For each sequence, the y-axis indicates SHAP importance value, and the x-axis represents positions relative to the TSS (-300 to +300 bp). Red dots and corresponding text annotations highlight three 6-mer tokens selected based on the highest absolute SHAP values, subject to two filtering criteria: tokens are only selected if they are not at the beginning of the sequence (position > 20) and are at least 25 nucleotides distant from any other selected token. Tokens are displayed by the order of SHAP importance, and their starting positions within the promoter window are indicated. Results are shown for two negative data generation strategies used during model training: **A.** positive-promoter-shuffled and **B.** random-non-promoter-fragments.

Organism	Positive-promoter-shuffled			Random-non-promoter-fragments		
	Character	min	max	Character	min	max
G.gallus	0.81	0.83	0.91	0.77	☆ 0.87	0.94
C.elegans	0.79	0.82	0.89	0.81	0.64	0.88
D.melanogaster	0.77	0.76	0.87	0.74	0.57	0.87
H.sapiens	0.82	☆ 0.90	0.96	0.84	0.71	0.91
M.musculus	0.72	☆ 0.85	0.96	0.69	0.52	0.92
M.mulatta	0.72	☆ 0.84	0.88	0.77	☆ 0.88	0.95
R.norvegicus	0.77	☆ 0.88	0.93	0.80	0.70	0.93
D.rerio	0.74	☆ 0.85	0.90	0.68	0.48	0.90

Fig. S7: Performance comparison (AUC) of character-level tokenizer relative to multi-character tokenizers, across eight organisms (rows), evaluated using two negative data generation strategies (columns): positive-promoter-shuffled and random-non-promoter-fragments. For reference, the minimum (min) and maximum (max) AUC values achieved by the four primary tokenization methods (taken from Figure 2) are shown to contextualize character-level performance. Statistically significant differences (FDR < 0.05, Benjamini-Hochberg corrected) where the *min* AUC value is superior to the character-level AUC value are marked with a white asterisk (*), placed next to the min value. The complete set of raw and adjusted p-values is provided in Table S3.B.

Method	Positive-promoter-shuffled		Random-non-promoter-fragments		Positive-promoter-shuffled		Random-non-promoter-fragments	
	No pretrain	With pretrain	No pretrain	With pretrain	No pretrain	With pretrain	No pretrain	With pretrain
	G.gallus				***C.elegans***			
BPE	0.80	☆ 0.83	0.91	☆ 0.94	0.79	0.82	0.82	0.85
WPC	0.84	☆ 0.87	0.91	☆ 0.94	0.84	☆ 0.87	0.85	☆ 0.88
Overlapping 6-mer	0.88	☆ 0.91	0.84	☆ 0.87	0.86	☆ 0.89	0.61	0.64
Non Overlapping 6-mer	0.82	0.85	0.91	☆ 0.94	0.85	☆ 0.88	0.77	☆ 0.80
	D.melanogaster				***H.sapiens***			
BPE	0.74	0.77	0.78	☆ 0.81	0.87	☆ 0.90	0.88	☆ 0.91
WPC	0.73	0.76	0.81	☆ 0.84	0.88	☆ 0.91	0.88	☆ 0.91
Overlapping 6-mer	0.77	☆ 0.80	0.54	0.57	0.91	☆ 0.94	0.68	0.71
Non Overlapping 6-mer	0.84	☆ 0.87	0.84	☆ 0.87	0.93	☆ 0.96	0.88	☆ 0.91
	M.musculus				***M.mulatta***			
BPE	0.83	☆ 0.86	0.88	☆ 0.91	0.83	☆ 0.86	0.92	☆ 0.95
WPC	0.90	☆ 0.93	0.89	☆ 0.92	0.85	☆ 0.88	0.91	☆ 0.94
Overlapping 6-mer	0.82	0.85	0.49	0.52	0.81	☆ 0.84	0.85	☆ 0.88
Non Overlapping 6-mer	0.93	☆ 0.96	0.87	☆ 0.90	0.85	☆ 0.88	0.90	☆ 0.93
	R.norvegicus				***D.rerio***			
BPE	0.85	☆ 0.88	0.89	☆ 0.92	0.82	0.85	0.86	☆ 0.89
WPC	0.87	☆ 0.90	0.89	☆ 0.92	0.84	☆ 0.87	0.87	☆ 0.90
Overlapping 6-mer	0.89	☆ 0.91	0.67	0.70	0.87	☆ 0.90	0.45	0.48
Non Overlapping 6-mer	0.90	☆ 0.93	0.90	☆ 0.93	0.86	☆ 0.89	0.86	☆ 0.89

Fig. S8: Performance comparison (AUC) of models trained with and without an initial pretraining step across tokenization methods. Shown are results for models trained directly on the fine-tuning data (no pretrain) versus models that included an initial pretraining step (with pretrain, corresponding to Figure 2). AUC values are reported for four tokenization methods (rows): BPE, WPC, overlapping 6-mer, and non-overlapping 6-mer, evaluated under two negative data generation strategies (columns): positive-promoter-shuffled and random-non-promoter-fragments, across eight organisms. The color gradient represents AUC values, with lighter colors indicating lower performance and darker colors indicating higher performance. Statistically significant differences (FDR < 0.05, Benjamini-Hochberg corrected) are marked with a white asterisk (*) placed next to the configuration (no pretrain/with pretrain) that achieved the higher AUC value. The complete set of raw and adjusted p-values is provided in Table S3.C.

Method	Positive-promoter-shuffled		Random-non-promoter-fragments		Positive-promoter-shuffled		Random-non-promoter-fragments	
	Short	Long	Short	Long	Short	Long	Short	Long
	G.gallus				***C.elegans***			
BPE	☆ 0.90	0.83	0.93	0.94	☆ 0.89	0.82	0.84	0.85
WPC	0.86	0.87	0.92	☆ 0.94	0.86	0.87	0.87	0.88
Overlapping 6-mer	0.80	☆ 0.91	0.89	0.87	0.87	0.89	0.71	0.64
Non Overlapping 6-mer	☆ 0.91	0.85	0.94	0.94	☆ 0.90	0.88	☆ 0.86	0.80
	D.melanogaster				***H.sapiens***			
BPE	0.82	0.77	☆ 0.84	0.81	0.91	0.90	0.90	☆ 0.91
WPC	0.75	0.76	0.84	0.84	0.90	☆ 0.91	0.91	0.91
Overlapping 6-mer	0.80	0.80	☆ 0.75	0.57	0.94	0.94	0.74	0.71
Non Overlapping 6-mer	0.86	0.87	0.87	0.87	0.93	☆ 0.96	0.93	0.91
	M.musculus				***M.mulatta***			
BPE	0.87	0.86	0.91	0.91	0.86	0.86	0.94	☆ 0.95
WPC	0.94	0.93	0.92	0.92	0.88	0.88	0.94	0.94
Overlapping 6-mer	0.85	0.85	☆ 0.73	0.52	0.84	0.84	0.88	0.88
Non Overlapping 6-mer	0.97	0.96	0.90	0.90	0.88	0.88	☆ 0.95	0.93
	R.norvegicus				***D.rerio***			
BPE	☆ 0.94	0.88	0.94	0.92	☆ 0.89	0.85	0.88	0.89
WPC	0.90	0.90	0.93	0.92	0.87	0.87	0.89	0.90
Overlapping 6-mer	0.91	0.91	0.74	0.70	0.82	☆ 0.90	☆ 0.78	0.48
Non Overlapping 6-mer	0.93	0.93	0.94	0.93	0.88	0.89	0.89	0.89

Fig. S9: Performance comparison (AUC) of promoter sequence lengths across tokenization methods. Shown are results for a shorter promoter window (301 bp, spanning positions -250 to +50 around the TSS) compared with the longer (601 bp, spanning positions -300 to 300) used in the primary analysis in Figure 2. AUC values are reported for four tokenization methods (rows): BPE, WPC, overlapping 6-mer, and non-overlapping 6-mer, evaluated under two negative data generation strategies (columns): positive-promoter-shuffled or random-non-promoter-fragments, across eight organisms. The color gradient represents AUC values, with lighter colors indicating lower performance and darker colors indicating higher performance. Statistically significant differences (FDR < 0.05, Benjamini–Hochberg corrected) are marked with a white asterisk (*), placed next to the window size that achieved the higher AUC value. The complete set of raw and adjusted p-values is provided in Table S3.D.
